# Supplementary material for: Chronic neuronal activation increases dynamic microtubules to enhance functional axon regeneration after dorsal root crush injury
Source: Nat Commun. 2020 Nov 30;11:6131. doi: 10.1038/s41467-020-19914-3 (PMC7705672; doi:10.1038/s41467-020-19914-3)
Supplement: Supplementary file 1 — Supplementary Information [file 41467_2020_19914_MOESM1_ESM.pdf]

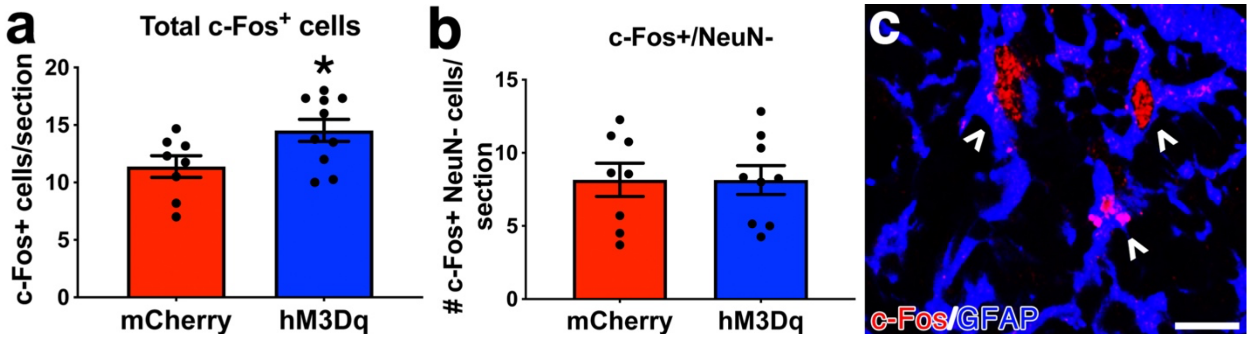

Supplemental Figure 1: Induction of c-Fos in non-neuronal cells. Twelve weeks after complete C4-T1 dorsal root crush injuries, ipsilateral median and ulnar nerves were isolated and electrically stimulated for 30 min. Animals were sacrificed 1 hr later. Spinal cord sections between C5 and C8 were processed for immunohistochemistry to visualize c-Fos (red) and NeuN (blue). Neuronal activation resulted in a significantly more cells expressing c-Fos (**a**). Some of these were $NeuN^-$ cells in white matter (**b**) that express GFAP (**c**, open arrowheads) and are presumably astrocytes. N=8 $mCherry^+$ animals and 10 $hM3Dq^+$ animals at 12 weeks. Mean ± SEM. Two-tailed unpaired t-test, *p=0.035. Scale bar: 10μm. Source data are provided as a Source Data file.
