# Supplementary material for: Chronic neuronal activation increases dynamic microtubules to enhance functional axon regeneration after dorsal root crush injury
Source: Nat Commun. 2020 Nov 30;11:6131. doi: 10.1038/s41467-020-19914-3 (PMC7705672; doi:10.1038/s41467-020-19914-3)
Supplement: Supplementary file 3 — Reporting Summary [file 41467_2020_19914_MOESM3_ESM.pdf]

nature research

Corresponding author(s): Veronica J. Tom

Last updated by author(s): Oct 23, 2020

Reporting Summary

Nature Research wishes to improve the reproducibility of the work that we publish. This form provides structure for consistency and transparency in reporting. For further information on Nature Research policies, see Authors & Referees and the Editorial Policy Checklist .

Statistics

For all statistical analyses, confirm that the following items are present in the figure legend, table legend, main text, or Methods section.

n/a	Confirmed	
☐	☒	The exact sample size (*n*) for each experimental group/condition, given as a discrete number and unit of measurement
☐	☒	A statement on whether measurements were taken from distinct samples or whether the same sample was measured repeatedly
☐	☒	The statistical test(s) used AND whether they are one- or two-sided<br>*Only common tests should be described solely by name; describe more complex techniques in the Methods section.*
☐	☒	A description of all covariates tested
☐	☒	A description of any assumptions or corrections, such as tests of normality and adjustment for multiple comparisons
☐	☒	A full description of the statistical parameters including central tendency (e.g. means) or other basic estimates (e.g. regression coefficient) AND variation (e.g. standard deviation) or associated estimates of uncertainty (e.g. confidence intervals)
☐	☒	For null hypothesis testing, the test statistic (e.g. *F*, *t*, *r*) with confidence intervals, effect sizes, degrees of freedom and *P* value noted<br>*Give P values as exact values whenever suitable.*
☒	☐	For Bayesian analysis, information on the choice of priors and Markov chain Monte Carlo settings
☒	☐	For hierarchical and complex designs, identification of the appropriate level for tests and full reporting of outcomes
☒	☐	Estimates of effect sizes (e.g. Cohen's *d*, Pearson's *r*), indicating how they were calculated

Our web collection on statistics for biologists contains articles on many of the points above.

Software and code

Policy information about availability of computer code

Data collection	Leica LasX, SlideBook 6
Data analysis	ImageJ 1.47V, GraphPad Prism 8

For manuscripts utilizing custom algorithms or software that are central to the research but not yet described in published literature, software must be made available to editors/reviewers. We strongly encourage code deposition in a community repository (e.g. GitHub). See the Nature Research guidelines for submitting code & software for further information.

Data

Policy information about availability of data

All manuscripts must include a data availability statement. This statement should provide the following information, where applicable:

- Accession codes, unique identifiers, or web links for publicly available datasets
- A list of figures that have associated raw data
- A description of any restrictions on data availability

All data supporting the findings of this study are available from the corresponding author upon reasonable request. The source data for Figures 1d, 2j, 3g, 5a-c, 6e-h, 7d-f, 8e-h, 9e-h, 10g, Supplemental Figure 1a-b are provided in the Source Data file.

Field-specific reporting

Please select the one below that is the best fit for your research. If you are not sure, read the appropriate sections before making your selection.

☒ Life sciences ☐ Behavioural & social sciences ☐ Ecological, evolutionary & environmental sciences

Life sciences study design

All studies must disclose on these points even when the disclosure is negative.

Sample size	Sample sizes were chosen based on previous experience and publications using similar methodology [e.g., Wu, D. et al. Expressing Constitutively Active Rheb in Adult Dorsal Root Ganglion Neurons Enhances the Integration of Sensory Axons that Regenerate Across a Chondroitinase-Treated Dorsal Root Entry Zone Following Dorsal Root Crush. Front Mol Neurosci 9, 49, doi:10.3389/fnmol.2016.00049 (2016) and Leo, L., Yu, W. & Baas, P. W. Using siRNA to study microtubule-related proteins in cultured neurons. Methods Cell Biol 131, 163-176, doi:10.1016/bs.mcb.2015.06.005 (2016).]
Data exclusions	No data were excluded from analysis.
Replication	All in vitro experiments were replicated three times. Animal experiments were conducted in two cohorts, with controls in each.
Randomization	Animals and neuron cultures were randomly assigned to treatment group.
Blinding	All experiments were analyzed while blinded to treatment group.

Reporting for specific materials, systems and methods

We require information from authors about some types of materials, experimental systems and methods used in many studies. Here, indicate whether each material, system or method listed is relevant to your study. If you are not sure if a list item applies to your research, read the appropriate section before selecting a response.

Materials & experimental systems

n/a	Involved in the study	
☐	☒	Antibodies
☒	☐	Eukaryotic cell lines
☒	☐	Palaeontology
☐	☒	Animals and other organisms
☒	☐	Human research participants
☒	☐	Clinical data

Methods

n/a	Involved in the study	
☒	☐	ChIP-seq
☒	☐	Flow cytometry
☒	☐	MRI-based neuroimaging

Antibodies

Antibodies used	Primary antibodies: c-Fos (Santa Cruz, sc-52); NeuN (Millipore, Clone A60, MAB377); NF-200 (Sigma, N0142); IB4 (Sigma, 217660); CGRP (Peninsula Labs, T-4032); BIII-tubulin (Sigma, T8660); mCherry (Abcam, ab8871); p-75 (Millipore, MAB5254); BIII-tubulin (Biolegend, PRB435P); acetylated tubulin (Sigma, T6793); tyrosinated tubulin (Sigma, TUB-1A2: T9028); p-S6 (Cell Signaling, 5364).<br><br>Secondary antibodies: Goat anti-mouse Alexa 488 (Invitrogen, A11001); Goat anti-mouse Alexa 594 (Invitrogen, A11005); Goat anti-rabbit Alexa 488 (Invitrogen, A11008); Goat anti-rabbit Alexa 594 (Invitrogen, A11012)
Validation	We personally validated the c-Fos antibody using a positive control (cervical spinal cord from an animal that whose median and ulnar nerves were electrically stimulated) and a negative control (an unstimulated animal). Furthermore, all antibodies used for immunohistochemistry are commercially available and were validated by the vendors. Abcam, Biolegend, Cell Signaling, Millipore, Peninsula Labs, Santa Cruz, and Sigma have standard analysis/validation processes for each lot of antibody that are described on their respective websites and/or product specification sheets.

Animals and other organisms

Policy information about studies involving animals; ARRIVE guidelines recommended for reporting animal research

Laboratory animals	Adult Wistar female rats (225g, about 12 weeks old) were used
Wild animals	No wild animals were used in these studies.
Field-collected samples	No field-collected samples were used in these studies.
Ethics oversight	This study was approved by the Drexel University Institutional Animal Use and Care Committee

Note that full information on the approval of the study protocol must also be provided in the manuscript.
